# Reply to: Comment on “Association of mean platelet volume with echocardiographic findings in patients with severe rheumatic mitral stenosis”

**DOI:** 10.15171/jcvtr.2019.56

**Published:** 2019-11-07

**Authors:** Naser Aslanabadi, Ahmad Separham, Leyla Valae Hiagh, Farid Karkon Shayan, Mehrnoush Toufan, Samad Ghaffari, Elgar Enamzadeh

**Affiliations:** ^1^Cardiovascular Research Center, Tabriz University of Medical Sciences, Tabriz, Iran; ^2^Students’ Research Committee, Tabriz University of Medical Science, Tabriz, Iran; ^3^Connective Tissue Diseases Research Center, Tabriz University of Medical Sciences, Tabriz, Iran

We thank Dr. Coban for his comment on our paper entitled “Association of mean platelet volume with echocardiographic findings in patients with severe rheumatic mitral stenosis”.^1^ In our study, venipuncture was done from antecubital vein and blood samples were collected in dipotassium ethylenediaminetetraacetic acid (EDTA) tubes. All hematologic indices were recorded by an automated blood cell counter (Sysmex KX-21N Automated Hematology Analyzer, Sysmex Corporation, USA). Platelet profile including MPV was measured within an hour from sampling to prevent EDTA-induced platelet changes.

Different instruments for measuring MVP could lead to variable MVP results as previously mentioned.^2,3^ Also, the time elapsed from venipuncture to MPV measurement can affect its results.^4,5^ Yet, standardization of MPV measurement needs large scale studies with sufficient power.^6^ Meanwhile, we used a routine measurement approach like most of previous studies.^7,8^ So, it seems unlikely that our MPV results be reported erroneously.^9^

## Competing interests

None.

## Ethical approval

Not applicable.
